# Mesoscale Recovery of Microglial and Neuronal Dynamics After Craniotomy Across Wide Cortex in Transgenic Mice

**DOI:** 10.1002/advs.202512192

**Published:** 2026-02-11

**Authors:** Guihua Xiao, Zhilei Wang, Pengchang Zheng, Jingyu Xie, Yangzhen Wang, Yun Chen, Zilin Wang, Ao Li, Minghuan Wang, Quanbo Ji, Can Gao, Di Yao, Lingbo Li, Jiangbei Cao

**Affiliations:** ^1^ Beijing National Research Center For Information Science and Technology Tsinghua University Beijing China; ^2^ Department of Automation Tsinghua University Beijing China; ^3^ Institute For Brain and Cognitive Sciences Tsinghua University Beijing China; ^4^ NMPA Key Laboratory For Research and Evaluation of Narcotic and Psychotropic Drugs Jiangsu Province Key Laboratory of Anesthesiology Jiangsu Province Key Laboratory of Anesthesia and Analgesia Application Xuzhou Medical University Xuzhou China; ^5^ Department of Anesthesiology The First Medical Centre, Chinese PLA General Hospital Beijing China; ^6^ Tsinghua‐Peking Center For Life Science Beijing China; ^7^ Department of Neurology Tongji Hospital Tongji Medical College Huazhong University of Science and Technology Wuhan China; ^8^ Hubei Key Laboratory of Neural Injury and Functional Reconstruction Huazhong University of Science and Technology Wuhan China; ^9^ Department of Orthopedics The First Medical Center Chinese PLA General Hospital Beijing China

**Keywords:** craniotomy, dynamics, mesoscale, microglia, neurons

## Abstract

Craniotomy is commonly used to access the brain for neurosurgical procedures or neural probe implantation. However, the dynamic structural and functional changes in microglia and neurons during postoperative recovery remain poorly understood. Here, we utilize mesoscale fluorescence imaging and multiple transgenic mouse models (Cx3cr1‐GFP, Thy1‐YFP, and Rasgrf2‐2A‐dCre/Ai148D) to longitudinally record the dynamic recovery of microglia and neurons across the wide cortex over 50 days after craniotomy. Our findings reveal that both neuronal and microglial structures and functions are significantly altered after surgery, yet their recovery to the first day after craniotomy follow distinct temporal patterns. Microglia exhibit the most rapid structural changes, reaching peak inflammatory response within approximately 10 days. Subsequently, neuronal structural fluorescence intensity peaks around 14 days post‐surgery, showing a strong positive correlation with microglial changes. Finally, neuronal functional dynamics are assessed using drifting grating visual stimulation, with functional modularity indices to quantify network integrity. We observe that functional modularity undergoes significant disruption, reaching its peak disruption at approximately 21 days post‐surgery. These findings provide new insights into the dynamic process following craniotomy and offer a novel perspective on neuroimmune interactions in traumatic conditions.

## Graphical abstract

This study employs longitudinal fluorescence imaging in transgenic mice to map post‐craniotomy cortical recovery. We identify distinct neuroimmune recovery phases: microglial structural inflammation peaks at ∼10 days, neuronal structural intensity peaks at ∼14 days and correlates with microglial activity, and functional network modularity is most disrupted at ∼21 days. This reveals a new temporal framework for post‐traumatic neuroimmune interaction.

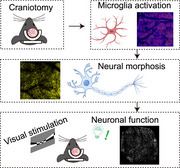

## 1 Introduction

Craniotomy is a fundamental neurosurgical procedure widely employed in clinical settings [1, 2], including intracerebral hemorrhage evacuation [3, 4], decompression of traumatic hematomas [5, 6], tumor resection [7], and implantation of microprobe devices [8, 9, 10]. Despite its essential clinical roles, craniotomy inherently induces complex neuroimmune responses [11, 12], significantly impacting postoperative neural recovery and long‐term outcomes. These immune responses modulate neuronal survival, synaptic plasticity, and the stability and integration of implanted neural interfaces. However, the spatiotemporal dynamics of post‐surgical inflammation, particularly its effects on microglial activation and neuron–glia interactions at single‐cell resolution, remain poorly characterized across the cortex. Understanding how craniotomy‐induced inflammation shapes glial reactivity and neuronal structure‐function relationships is essential for both fundamental neuroscience and translational applications.

Cranial window surgery would induce inflammation and trigger microglial activation, scar tissue formation, and alterations in neuronal function and connectivity [13, 14]. The recovery time for these alterations varies, typically stabilizing between 4 to 21 days post‐surgery [12, 15]. Two‐photon microscopy was used to track the activation of microglia and the dynamics of neurons over time. For example, extensive microglia activation within cranial window preparations was observed [12], noting changes in cell structure and density. In contrast, astrocytes remained inactive and did not express GFAP even after 4–5 weeks following the surgery [16]. Neuronal kinetics, including dendritic spine number and filopodia turnover, were also impacted by chronic cranial window implantation, as demonstrated in observing synaptic changes [12, 17].

These independent findings highlight the importance of understanding both glial activation and neuronal dynamics to assess the impact of cranial window surgeries on brain function. However, most research has focused on examining a very small range of cellular or molecular changes to identify differences in cellular structure. There is no study assessing the functional dynamics across the wide cortex and how cellular interactions evolve post‐injury and influence functional outcomes such as sensory processing. It has been largely limited by the capabilities of observational techniques. Recent advances in imaging technologies now enable dynamic observation at single‐cell resolution across wide cortex in living animals. For example, two‐photon synthetic aperture microscopy (2pSAM) [18, 19, 20, 21] and digital adaptive optics scanning light‐field mutual iterative tomography (DAOSLIMIT) system [22] allow for sub‐cellular high‐resolution within a field of view (FOV) extending hundreds of micrometers. Meanwhile, mesoscale optical imaging (RUSH 3D) allows recording of neural activities across a large FOV at single‐cell resolution [23, 24, 25, 26].

To achieve a detailed characterization of glial and neuronal dynamics across wide cortex, we utilized 2pSAM and RUSH 3D microscopy system to characterize the dynamics of microglial (Cx3cr1‐GFP) and neuronal morphology (Thy1‐YFP) in the brain following craniotomy, and assessed the neuronal functional (Rasgrf2‐2A‐dCre/Ai148D) responses to visual stimuli. The results showed that microglia began to activate immediately after surgery, with changes in morphology characterized by the gradual elongation of cell processes, an increase in fluorescence intensity, and an expansion in 3D volume. This activity peaked around the 10th day post‐surgery, after which it declined and stabilized. Neuronal fluorescence intensity also showed an upward trend, though it was slower than that of the glial cells, with a slight decrease observed during the first couple of days. We observed a distinct decline in neuronal function in response to visual stimuli during the early stages after craniotomy, as evidenced by a reduced coding rate and functional modularity. By combining advanced imaging techniques with genetically modified animal models, this work provides a comprehensive overview of cellular changes in both glial and neuronal dynamics across the wide cortex post‐surgery. These findings contribute to our understanding of the long‐term effects of craniotomy on the brain, highlighting the complex interactions between structural integrity and functional response.

## 2 Results

### 2.1 Experimental Paradigm and Mouse Models

**FIGURE 1:**
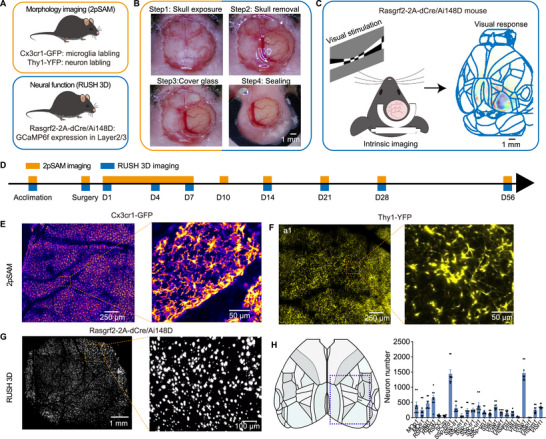
Experimental Paradigm and Mouse Models. (A) Mouse models used in the study: microglia labeled with Cx3cr1‐GFP, neurons structurally labeled with Thy1‐YFP (both imaged by 2pSAM microscopy), and neuronal calcium signaling at layer 2/3 labeled with Rasgrf2‐2A‐DCre/Ai148D (imaged by RUSH 3D microscopy). (B) Illustration of the craniotomy surgical procedure. (C) Diagram depicting intrinsic signal maps aligned to the Allen Brain Atlas. (D) Schematic of the experimental paradigm. Timepoints in yellow indicate 2pSAM imaging sessions; those in blue indicate RUSH 3D imaging sessions. (E–G) Representative images showing microglia (E), neuronal structure (F), neuronal calcium signaling (G), along with enlarged views of regions outlined in yellow. (H) Field‐of‐view indicated by a purple rectangle, with corresponding neuron counts distributed across wide cortex in 9 mice.

To study the dynamics of neurons and microglia in the cerebral cortex following craniotomy, we used several transgenic mouse models (Figure 1A). Microglia were visualized using Cx3cr1‐GFP mice, which express GFP in microglial cells, allowing for clear observation of their morphology. Neuronal structures were imaged in Thy1‐YFP mice, which provide broad labelling of neurons throughout the brain. This line primarily labels subsets of projection neurons, such as pyramidal neurons, but the labeling is not entirely restricted to this single neuronal subtype [27]. To monitor real‐time neuronal activity, we employed hybrid Rasgrf2‐2A‐dCre/Ai148D mice, which express the genetically encoded calcium indicator GCaMP6f specifically in layer 2/3 cortical neurons. All mice underwent a standard craniotomy procedure, commonly used for in vivo imaging. This involved exposing the skull, carefully removing a section while preserving the dura mater, and replacing it with a glass window, followed by standard wound closure (Figure 1B). The procedure was highly effective, with a success rate exceeding 80% when performed by experienced technicians. Mice that experienced significant complications—such as severe intraoperative bleeding—or those that failed to maintain good health or complete imaging sessions were excluded from the study as indicated in Figure S1.

Accurate brain map alignment is essential for wide‐field neuronal imaging, particularly when comparing functional differences across brain regions. This is especially critical in experiments involving sensory stimulation. To achieve precise alignment, we incorporated endogenous signal‐based imaging to assist with map registration. By repeatedly presenting visual stimuli to the mice, we evoked a widespread response in the visual cortex, which allowed us to clearly define the boundaries of the visual area (Figure 1C). This enabled accurate alignment of the standard Allen Brain Atlas with the neuronal activity maps obtained from our imaging data.

To fully document the dynamic process following craniotomy, imaging was conducted from the day after surgery and continued until recovery to the first day after craniotomy was complete (Figure 1D). The first imaging session took place 1 day after surgery, with subsequent sessions conducted at approximately the same time each day to maintain a consistent 24‐h interval. We tracked changes in both immune and neuronal signals over a 56‐day period. Since the immune response is typically most active during the first week post‐surgery, we increased the sampling frequency during this initial phase to better capture rapid and dynamic changes in immune activity. Structural imaging was performed using our in‐house 2pSAM microscope, which offers high resolution suitable for visualizing cellular structures, including microglia (Figure 1E) and neuronal structures (Figure 1F). Apparent differences in vasculature size between Cx3cr1‐GFP and Thy1‐YFP images arise from biological variation between individual mice, differences in imaging depth, and indirect visualization of blood vessels rather than differences in the field of view. Dynamic calcium activity was monitored with the RUSH 3D system (Figure 1G), which provides a wide field of view ideal for capturing large‐scale neuronal activity across 22 brain regions (Figure 1H).

### 2.2 Long‐Term Tracking of Microglia Structural Dynamics

Microglia were chosen to monitor immune responses due to their essential role as the primary resident immune cells in the central nervous system (CNS). They rapidly detect and respond to cellular damage, pathogens, or injury, making them critical for maintaining brain homeostasis. During and after open‐skull surgery, microglia become activated, acting as the first line of defense by detecting and reacting to changes in their local environment. Visualizing the dynamics of microglia post‐surgery provides valuable insights into their roles in neuroinflammation, tissue repair, and overall brain health following surgical trauma.

**FIGURE 2:**
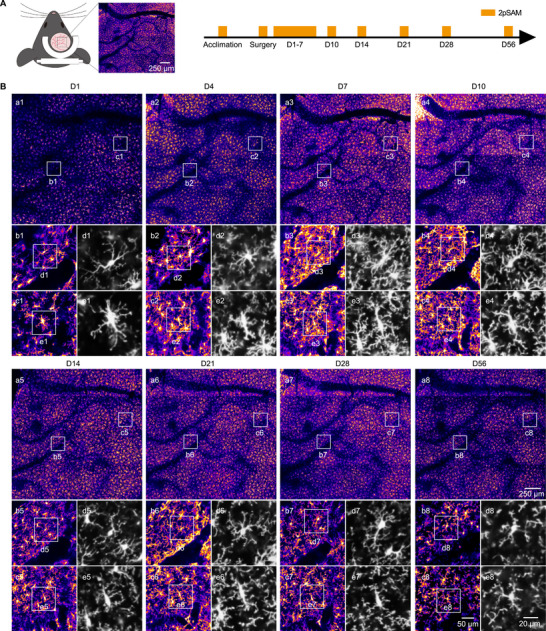
Long‐term in vivo tracking of microglial dynamics using 2pSAM. (A) A representative image of large‐scale 2pSAM image (left) and in vivo 2pSAM imaging timeline in Cx3cr1‐GFP mice (right). (n = 3 mice) (B) Representative images of microglial structure observed in 4 × 4 scanning of 2pSAM imaging (a1‐a8), with enlarged views of microglia morphology at different time points post‐surgery (b1–b8, c1–c8). The same individual microglia were longitudinally tracked as shown in d1–d8 and e1–e8.

To examine these structural dynamics over an extended period, microglia were tracked using 2pSAM imaging (Figure 2A). While microglia display continuous, rapid process motility on a second‐to‐minute timescale [28, 29], these small, dynamic movements occur constantly in the background. In contrast, more pronounced and quantifiable morphological changes—such as branch restructuring, major shifts in process orientation, or soma migration—typically emerge gradually over the course of hours to days [30]. Therefore, we employed a 4 × 4 scanning mode to effectively capture cellular dynamics within a large field‐of‐view (FOV) of approximately 2.1 mm × 2.1 mm. Imaging FOVs were carefully aligned with previous sessions to consistently track identical cell populations over time. Representative imaging results from one mouse illustrated typical structural evolution from postoperative day 1 to day 56 (Figure 2B, Supplementary video S1). By closely examining microglia near blood vessels as well as regions lacking vasculature, we observed clear morphological transitions. Initially, microglia presented rounded cell bodies with numerous cellular processes. As the dynamic period progressed, the cell bodies gradually elongated, and density of processes increased. However, starting around 2 weeks post‐surgery, the number of processes began to decline, and microglia progressively reverted toward a more resting, baseline morphology.

**FIGURE 3:**
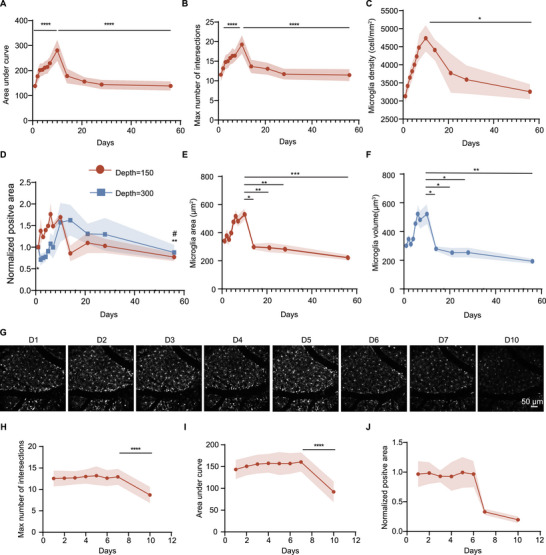
Statistical analysis of long‐term in vivo tracking of microglial dynamics. (A,B) Quantitative analysis of microglia by Sholl analysis post‐surgery by 2D images (n = 90 cells from 3 mice). (C,D) Quantitative analysis of microglial density and normalized positive area post‐surgery by 2D images (n = 3 mice). (E,F) Quantitative analysis of microglial surface area and volume post‐surgery after 3D reconstruction (n = 3 mice). (G–J) Representative images (G) and quantitative results of Sholl analysis (H, I) and normalized positive area (J) after skull thinning and transparency procedures. (Scale bar, 50 µm. n = 45 cells from 3 mice). RM One‐way ANOVA, Tukey's *post hoc* correction in (C, D, E, F, J). Friedman test with Dunn's *post hoc* correction in (A, B, H, I). *p < 0.05, **p < 0.01, ***p < 0.001, ****p < 0.0001. **p < 0.01 vs Peak‐timepoint for depth = 150 µm, and #*p* < 0.05 vs Peak‐timepoint for depth = 300 µm in (D).

We further quantified microglial dynamics using both 2D measurements and 3D structural reconstructions. Sholl analysis, a well‐established method for assessing microglial morphology, revealed that microglial process complexity initially increased, peaked, and subsequently declined over the recovery period (Figure 3A,B), suggesting a transition from a relatively resting state to a hyper‐ramified phase following craniotomy. In addition, we quantified microglial cell density and observed a similar pattern of initial increase followed by gradual reduction (Figure 3C). Analysis of Cx3cr1 fluorescence intensity and positive area across cortical depths ranging from 150 to 300 µm consistently revealed comparable trends (Figure 3D, Figure S2A). Furthermore, we reconstructed individual microglial cells and performed automated quantification of surface area (Figure 3E) and cell volume (Figure 3F) using Imaris software. These reconstructions indicated a postoperative increase in both surface area and volume during the early phase, which later plateaued as microglia stabilized into a steady morphological state. To further validate these in vivo findings, we also performed ex vivo Iba1 immunostaining at key timepoints (pre‐surgery baseline, day 10, day 28). The ex vivo results were consistent with the in vivo imaging, supporting the observed morphological changes (Figure S2B,C). Collectively, these findings clearly demonstrated that the intracranial inflammatory response, represented by microglial cells, initiated gradually after craniotomy, peaked around day 10, and subsequently subsided. This process was primarily characterized by morphological transition to a hyper‐ramified state and an increase in cell density.

Since skull transparency methods are believed to induce a milder inflammatory response compared to open‐skull fenestration, we directly compared microglial dynamics between these surgical techniques. Skull thinning and transparency procedures were performed and analyzed alongside the open‐skull method. Quantitative analysis of microglial responses over the first 7 days were minimal and statistically non‐significant (Figure 3G–J, Figure S2D), although a significant reduce due to skull regeneration occurred after day 7, as indicated by calculated the index of vessel contrast (Figure S2E). This stood in stark contrast to the robust and escalating activation observed in the open‐skull craniotomy group during the same period, suggesting a reduced inflammation when the skull is preserved, though imaging duration was limited due to the eventual occlusion.

### 2.3 Visualization of Neural Morphology Dynamics After Surgery

**FIGURE 4:**
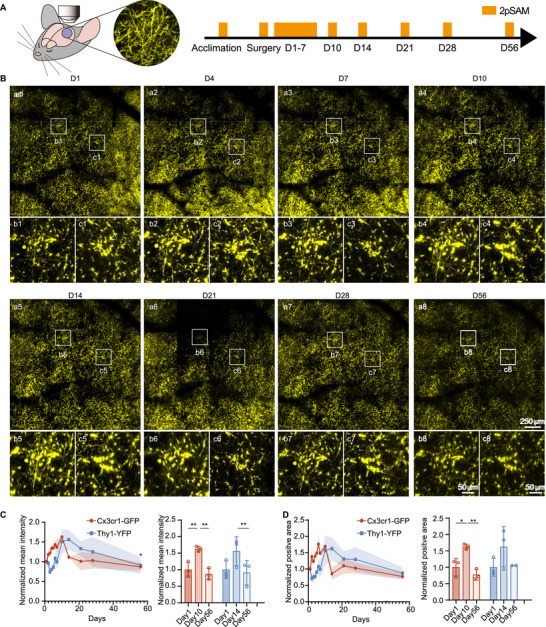
Long‐term in vivo tracking of neuronal dynamics using 2pSAM. (A) In vivo 2pSAM imaging timeline in Thy1‐YFP mice. (n = 3 mice) (B) Representative images of neuron structure observed in 4*4 scanning of 2pSAM imaging (a1–a8), with enlarged views of neuron morphology at different time points post‐surgery (b1–b8, c1–c8). Scale bar, 250 and 50 µm. (C) Comparison of normalized mean fluorescence intensity between microglial (orange) and neuronal (blue) morphology (left). The corresponding statistical analysis compares intensity values at day 1, 10, and 56 for microglia, and at day 1, 14, and 56 for neurons (right). (D) Comparison of normalized positive fluorescent area between microglial (orange) and neuronal (blue) morphology over time (left). The corresponding statistical comparison was performed across day 1, 10, and 56 for microglia, and day 1, 14, and 56 for neurons (right). Statistical significance was determined using RM one‐way ANOVA with Tukey's *post hoc* correction (*p < 0.05, **p < 0.01; n = 3 mice).

Inflammatory responses following surgery can potentially impact neuronal structures. To investigate the effects of fenestration surgery on neurons, we used Thy1‐YFP transgenic mice and applied the same imaging protocol previously utilized for microglia (Figure 4A). Neuronal morphology was also captured using two‐photon microscopy with a 4 × 4 scanning block method, allowing a large FOV to be acquired. Representative neuronal structure imaging results from one mouse illustrate typical structural evolution from postoperative day 1 to day 56 (Figure 4B, Supplementary Video S2). Enlargement of selected regions enabled clear visualization of morphological changes in neuronal cell bodies over time.

To quantitatively assess these changes, we measured both the average fluorescence intensity and the area of positive fluorescent signals. Neuronal fluorescence intensity remained low during the first 2–3 days after surgery, then began to increase significantly, peaking around day 14. Afterward, it gradually decreased and stabilized (Blue in Figure 4C, left). Statistical analysis confirmed that fluorescence intensity was highest at day 14 and returned to baseline by day 56, based on measurements from 3 mice (Blue in Figure 4C, right). The positive fluorescent area followed a similar temporal pattern (Blue in Figure 4D). We hypothesized that these observed changes in Thy1‐YFP signal might be associated with aberrant morphological alterations in dendritic spines. To test this, we performed ex vivo brain slice imaging in Thy1‐YFP transgenic mice and conducted quantitative analysis of dendritic spines. Our results showed that, compared to the non‐surgical baseline group, spine density was significantly increased at day 10 post‐craniotomy, with a predominance of spines exhibiting immature‐like morphological features. By day 28 post‐craniotomy, both the density and the compositional proportion of dendritic spines had recovered to levels comparable to baseline (Figure S3A–E). In addition, the mean fluorescence intensity and positive area in the fixed tissue at day 0, 10, and 28 were quantified, and the temporal trend of these ex vivo measurements closely mirrored the dynamics observed in our in vivo imaging (Figure S3F,G). In summary, these data indicated that craniotomy triggers a transient and dynamic reorganization of dendritic spines, suggesting a remarkable capacity for synaptic recovery following surgical intervention.

To further understand the dynamics of the neural response, we compared neuronal changes with those of microglia over the same time course. Both cell types exhibited broadly similar trends in response to surgical trauma. However, the increase in neuronal fluorescence intensity lagged slightly behind the activation of microglia. Specifically, microglial intensity peaked at approximately day 10, while neuronal intensity reached its maximum around day 14. These differences in peak intensities were statistically significant when compared to day 1 and day 56. The trends in positive fluorescent area were consistent with the intensity data for both neurons and microglia.

In summary, both microglia and neurons are significantly influenced by surgical trauma. Microglia respond rapidly, whereas neuronal responses are delayed but follow a similar trajectory. Over time, both cell types gradually return to their baseline states. We speculate that the early and rapid microglial activation after surgery may contribute to the delayed structural changes observed in neurons.

### 2.4 Characterization of Neuronal Functionality

**FIGURE 5:**
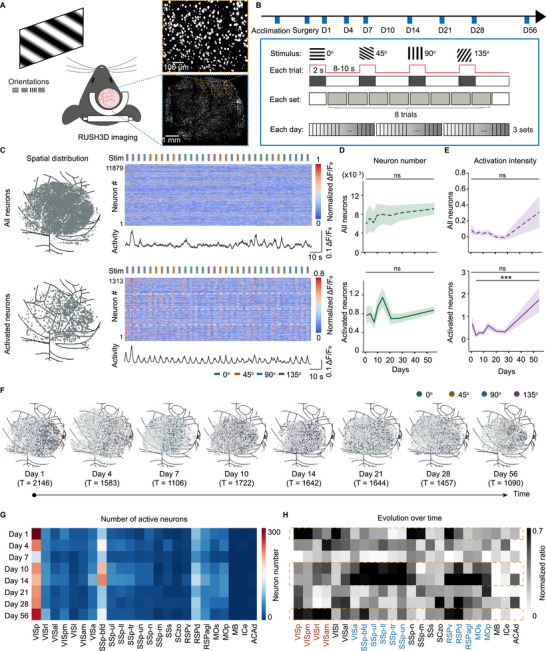
Neuronal population dynamics after craniotomy. (A) Schematic of the RUSH 3D imaging platform used to record neuronal activity in response to four different orientations of visual stimuli. (B) Experimental paradigm for visual stimulation, with each orientation presented eight times per stimulus set. (C) Spatial distribution of all detected neurons (top) and stimulus‐responsive neurons (bottom), along with representative single‐cell traces and population‐averaged activity patterns. (D) Temporal dynamics of the total number of detected neurons (top) and stimulus‐responsive neurons (bottom) from day 1 to day 56 post‐surgery (mean ± s.e.m, n = 4 mice). (E) Temporal changes in neuronal activity intensity for all neurons (top) and stimulus‐responsive neurons (bottom) over the same period (mean ± s.e.m, n = 4 mice). (F) Spatial distribution of stimulus‐responsive neurons across the cortex at different time points. (G) Quantification of stimulus‐responsive neuron distribution across distinct brain regions (averaged across mice, n = 4 mice). (H) Normalized distribution of stimulus‐responsive neurons across cortical regions over time (averaged across mice, n = 4 mice). Statistical analysis: Friedman test and Dunn's *post hoc* test with Bonferroni correction in (D and E), n = 4 mice. * *p* < 0.05, ** *p* < 0.01, *** *p* < 0.001, **** *p* < 0.0001, ns – not significant (p > 0.05). Please refer to the Source Data file for details on more statistics, p‐values, and other relevant information.

To evaluate neuronal functionality across the cortex after craniotomy, we implemented a visual stimulation paradigm combined with large‐scale calcium imaging. Drifting gratings at four orientations (0°, 45°, 90°, 135°) were presented in randomized order over a 20‐min session while calcium activity was recorded from over 10 000 neurons using the RUSH 3D imaging system (Figure 5A). Each session included three stimulus sets, with each orientation repeated eight times per set to reduce variability. Individual stimuli were shown for 2 s, followed by an 8–10 s inter‐stimulus interval (Figure 5B). Mice underwent habituation training prior to surgery, and recordings were conducted at designated postoperative time points.

We analyzed both the overall neuronal population (Figure 5C, top) and stimulus‐responsive subpopulations (Figure 5C, bottom), focusing on their spatial distribution and activity patterns. Stimulus‐activated neurons were sparsely distributed and comprised a relatively small fraction of the total population. Among them, some exhibited orientation‐selective responses, while others responded to multiple orientations. Compared to the overall population, the averaged activity traces of stimulus‐responsive neurons demonstrated stronger and more distinct tuning properties. To further assess neuronal dynamics, we quantified changes in neuron numbers across multiple mice. The number of both all detected neurons and stimulus‐activated neurons (Figure 5D) showed fluctuations in the days following surgery, gradually stabilizing at later time points. In contrast, the amplitude of neuronal activity (Figure 5E) remained relatively stable throughout the recovery period, except for a minor fluctuation observed at day 56 (comparison of stimulus‐activated neurons between day 4 and day 56: Friedman's χ^2^ (7) = 30.81, adjusted p = 0.0007; see Source Data file). Spatial mapping across recovery stages revealed dynamic changes in the distribution of responsive neurons (Figure 5F). Most of stimulus‐responsive neurons were widely distributed in the visual cortex with fewer in the other cortex.

Statistical analysis across multiple animals (Figure 5G) revealed that the majority of stimulus‐responsive neurons were located in the primary visual cortex (VISp), followed by the secondary somatosensory area (SSp‐bfd) and the retrosplenial cortex (RSPd). Temporal profiling of normalized neuron counts across cortical regions (Figure 5H) showed an initial decline in responsive neurons shortly after surgery, followed by a gradual re‐expansion across distinct areas. Interestingly, at both day 1 and day 56 post‐surgery, the majority of stimulus‐responsive neurons were concentrated in visual regions, including VISp, VISpl, VISli, and VISl. In contrast, during the intermediate stages (days 10 and 14), a larger proportion of responsive neurons emerged in somatosensory and retrosplenial areas. These results indicate region‐specific dynamics in cortical functional recovery to the first day after craniotomy, with transient shifts in the distribution of sensory processing activity over time.

Our results show that visual stimuli elicit early responses in the SSp‐bfd and RSPd regions, particularly on days 10 and 14, when input from the visual cortex remains limited. Recent studies have demonstrated that the retrosplenial cortex (RSC) receives feedback projections from higher‐order visual areas and the thalamus, allowing it to process visual information even in the absence of strong input from V1 [31, 32]. SSp‐bfd is also implicated in cross‐modal integration, with anatomical connections to VISp suggesting that visual signals may propagate through broader cortical circuits. In addition, behavioral states such as heightened arousal or movement can enhance visual responses by increasing cortical excitability, potentially contributing to the observed activity in SSp‐bfd and RSPd^32^. These findings indicate that both regions can respond to visual input despite reduced direct cortical drive. While prior studies have hinted at possible mechanisms underlying visually evoked responses in SSp‐bfd and RSPd, the early re‐emergence of neural activity in these areas on days 10 and 14 remains insufficiently understood. Our population‐level recordings reveal that both regions exhibit early activation during the post‐craniotomy period, providing a basis for future investigation into the circuit‐level mechanisms supporting this recovery.

### 2.5 Delayed Functional Recovery of Neurons Relative to Morphological Changes

**FIGURE 6:**
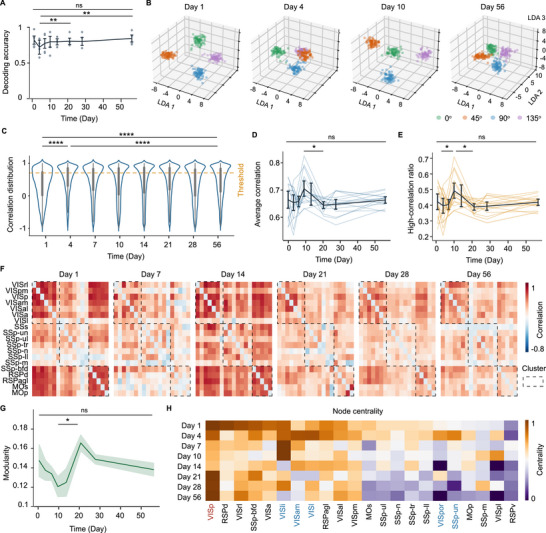
Neural functional connectivity changes during recovery to the first day after craniotomy. (A) The decoding accuracy changes from day1 to day 56 (mean ± s.e.m, n = 4 mice). (B) Linear discriminant analysis (LDA) was used to project high‐dimensional neuronal activity into a low‐dimensional space associated with four orientation stimuli. (C) Distribution of all correlations in the neuronal functional network. Functional connections with correlation coefficients exceeding 0.8 were classified as high‐correlation edges. (D) Changes in the average functional connectivity of the neuronal network (mean ± s.e.m, n = 4 mice). (E) Proportion of connections with a correlation greater than 0.8 among all connections (mean ± s.e.m, n = 4 mice). (F) Functional connectivity changes at the brain region level, with functional clustering derived from the first day post‐operation parcellation. (G) Modularity of the functional network measuring the prominence of functional clusters (mean ± s.e.m, n = 4 mice). (H) Node centrality within the functional network, distributed across different brain regions (averaged across mice, n = 4 mice). Statistical analysis: Friedman test and Dunn's *post hoc* test with Bonferroni correction in (A and G); Kolmogorov‐Smirnov test in (C); RM One‐way ANOVA with Tukey's *post hoc* correction in (D and E); n = 4 mice. * *p* < 0.05, ** *p* < 0.01, *** *p* < 0.001, **** *p* < 0.0001, ns – not significant (p > 0.05).

We evaluated the functional recovery of neural activity using low‐dimensional representation and functional network analysis. Visual function was assessed based on decoding performance and the structure of low‐dimensional representations of visually evoked activity. To quantify visual discrimination, we measured the decoding accuracy of neuronal population responses to drifting grating stimuli at different orientations using a support vector machine (SVM) classifier (Figure 6A). Decoding accuracy was tracked from day 1 to day 56 post‐surgery. Despite the surgical intervention, the decoding accuracy remained high overall, with a transient decrease observed around days 4 and 7 (comparison between day 4 and day 56: Friedman's χ^2^ (7) = 56.94, adjusted p = 0.0015), indicating a temporary disruption in visual discrimination selectivity. Performance returned to baseline levels thereafter. In parallel, we applied linear discriminant analysis (LDA) to project high‐dimensional population activity onto a low‐dimensional space associated with visual stimulus identity (Figure 6B). This analysis revealed that low‐dimensional representations of different grating orientations became less distinguishable on day 4, but gradually recovered and remained stable through day 56. These findings suggest an initial decline followed by restoration of visual discrimination at the population level.

Neurons form interconnected networks through interaction and collaboration to achieve various functions. Functional connectivity quantifies the strength of these interactions between individual neurons or brain regions (Figure 6C–E), with higher connectivity reflecting a more integrated and coordinated network. To assess functional recovery, we calculated Pearson correlation coefficients between neuronal units and compared them across time points. On day 1 post‐surgery, the distribution of correlation values was broad, whereas later time points showed an increased proportion of strong correlations (Figure 6C). Statistical analysis revealed a slight reduction in functional connectivity from day 1 to day 4, followed by a marked increase from day 4 to day 10 (Figure 6D). A modest decline was observed from day 10 to day 21. Given the importance of strong connections in maintaining network function, we further quantified the proportion of connections with correlation values above 0.8, which followed a similar trend (Figure 6E). These results suggest that, during the post‐craniotomy recovery period, global neuronal coordination initially declined, then recovered, and gradually stabilized over time. This indicates that during the post‐craniotomy recovery period, the global coordination of neuronal activity underwent a transient decline, followed by a subsequent recovery, before eventually reaching a state of progressive stabilization.

We further examined functional connectivity at the brain‐region level to investigate interactions between distinct cortical areas. Figure 6F showed the brain‐region‐level functional connectivity across multiple regions, which grouped into three clusters according to the connectivity network of the first post‐surgery. This cluster structure became less distinct between days 7 and 14, but gradually re‐emerged between days 21 and 56. To quantify this pattern, modularity is a property of functional connectivity networks that quantifies the extent to which the network can be partitioned into clusters with stronger internal than external connections, compared to a random null model. Statistical analysis revealed a gradual decrease in modularity from days 1 to 10 post‐surgery, followed by a significant increase between days 10 and 21 (Friedman's χ^2^ (7) = 15.73, adjusted p = 0.0461) (Figure 6G). A higher modularity value indicates a more distinct community structure, characterized by dense intra‐community connections and sparse inter‐community links. Consequently, high modularity signifies that the functional network is organized into several well‐segregated sub‐networks, for example, VIS sub‐network and RSP sub‐network in Figure 6F. By day 56, modularity had returned to a level similar to day 1.

Finally, degree centrality was analyzed as a key property of functional connectivity networks, reflecting the relative importance of each node. It is defined by the number of direct connections a node has with other nodes in the network (Figure 6H), with higher degree centrality indicating greater potential influence within the functional architecture. The primary visual cortex (VISp) exhibited elevated degree centrality during days 1–4 and days 21–56 post‐surgery (Figure 6H). In contrast, from days 4 to 10, regions such as the primary somatosensory cortex (SSp‐un) and several visual areas (including VISl, VISli, VISam, and VISpor) showed increased centrality, suggesting their prominent roles in maintaining network integration and facilitating functional recovery during this intermediate phase.

### 2.6 Interaction of Microglia and Neurons in Morphology and Function

**FIGURE 7:**
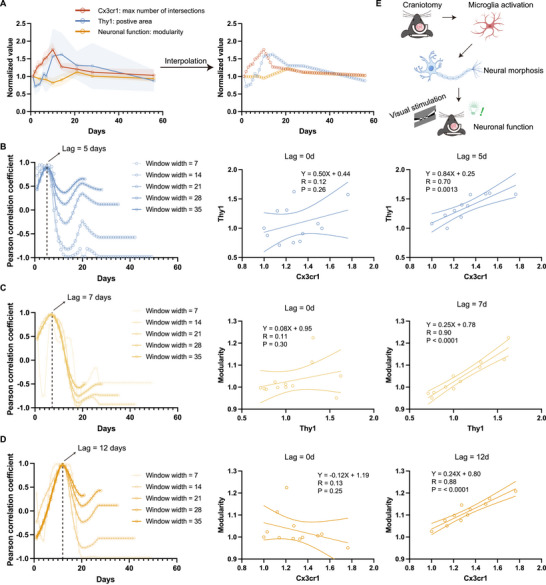
Dynamic correlations between microglial morphology, neuronal morphology, and neuronal function following open‐skull surgery. (A) Data linear interpolation was applied to cross‐day multimodal data (left), generating a continuous dataset with dynamically changing values over 56 consecutive days (right). (n = 3 mice for each group) (B) Pearson correlation analysis was performed when Cx3cr1‐GFP intensity window was fixed, while Thy1‐YFP intensity window was slide along time (from day 1, width = 7, 14, 21, 28, 35, respectively). The highest correlation appeared at a 5‐day lag (left). Cx3cr1 and Thy1 intensity at different timepoints exhibited low relation (middle), but showed a robust positive correlation at a 5‐day lag (right, R = 0.70, p = 0.0013). (C) Similar correlation analysis between Thy1‐YFP intensity and modularity of neural functional network, showing the highest correlation at a 7‐day lag (R = 0.90, *p* < 0.0001). (D) Similar correlation analysis between Cx3cr1 intensity and neural modularity, with a strong correlation at a 12‐day lag (R = 0.88, *p* < 0.0001).

To gain deeper insights into the dynamic relationship between the structural and functional dynamics of microglia and neurons following open‐skull surgery, we simultaneously analyzed the variation of microglial structure, neuronal structure, and neuronal function. Due to the limited number of data points, we first applied linear interpolation to cross‐day multimodal data [33, 34, 35], generating a continuous dataset with dynamically changing values over 56 consecutive days (Figure 7A). We then examined the correlations between these variables. Our findings indicated that microglial and neuronal intensity exhibit the highest correlation (R = 0.70) at a 5‐day lag, suggesting that microglial morphology changes precede neuronal morphological changes (Figure 7B). Additionally, neuronal function and morphology showed the highest correlation (R = 0.90) at a 7‐day lag, indicating that neuronal functional modulation follows structural changes (Figure 7C). Furthermore, we observed a robust correlation (R = 0.88) between microglial morphology and neuronal function at a 12‐day lag (Figure 7D). These results suggested that open‐skull surgery, as an invasive procedure, impacts both neuronal and immune cell dynamics in the brain (Figure 7E). Microglial responses peak and stabilize first, followed by the gradual increase in neuronal structural fluorescence intensity, while neuronal functional recovery to the first day after craniotomy is the most prolonged process.

## 3 Discussion

In conclusion, this study provides valuable insights into the dynamics of microglia and neurons following craniotomy. By leveraging transgenic animal models and advanced microscopy techniques, we tracked both morphological and functional changes in the brain over time. Our results showed that microglial activation occurred rapidly after surgery, peaking around day 10 and gradually returning to baseline. In contrast, neuronal activity exhibited a delayed increase in fluorescence intensity, accompanied by a transient decline in functional responses to visual stimuli, as reflected by reduced decoding accuracy. Neuronal functional correlation peaked around day 14, with recovery to the first day observed by approximately day 21. However, neuronal modularity peaked later, around day 21, representing the most affected and delayed response. Together, these findings highlight the intricate interplay between glial and neuronal dynamics, advancing our understanding of the long‐term impacts of craniotomy on brain function. This work lays a foundation for future studies aimed at uncovering the mechanisms of brain recovery and developing strategies to mitigate the effects of surgical interventions on neural performance.

As craniotomy is a widely used procedure in neuroscience for accessing the brain and enabling long‐term imaging [36, 37], our study provides important guidance for researchers aiming to understand recovery dynamics in such experimental models. Since glial cell activation can influence the interpretation of neural function [23, 38], we investigated the temporal relationship between glial activation and neuronal function during the postoperative recovery phase. Furthermore, understanding glia–neuron interactions is particularly valuable for studies involving chronic neural recordings [39, 40], as these interactions may confound measurements of neuronal activity, especially in long‐term imaging paradigms. Based on the observed neuronal function, a 1‐month postoperative period appears sufficient for mice to reduce surgery‐induced effects. We observed no statistically significant differences between day 28 and day 56 in measures of microglial activation (Figure 3B–E), neuronal structure (Figure 4C,D), and neuronal function (Figure 6), indicating that an extended observation period beyond 4 weeks did not reveal further major changes. Similarly, Koletar group [41] found that microglial and astrocytic activation begin resolving by ∼4 weeks post‐cranial window surgery in rats. Liu group [42] used a 2–4‐week recovery period after cranial window implantation for imaging and behavioral tests. Tournissac group [43] recommend ∼2 weeks recovery before imaging begins. Meanwhile, review by Cramer group [13] also notes that many protocols adopt 2–4 weeks as a standard recovery interval. It should be noted that our study does not include longitudinal pre‐craniotomy measurements within the same animals; therefore, the term “baseline” refers to the first day after craniotomy (Day 1), which serves as the reference point for subsequent comparisons. Consequently, the term “recovery” is used to describe a relative return toward lower levels of microglial activation or functional disruption compared with this post‐craniotomy baseline, rather than an absolute restoration to pre‐craniotomy conditions. In this study, we did not capture the full morphological trajectory of microglia from resting to fully recovered states. Following fenestration‐induced damage, microglia rapidly become activated compared to neurons [44, 45], and by the time imaging began, they were already in an activated state. Notably, their morphology did not fully return to baseline over time, suggesting that microglial activation may involve long‐lasting or potentially irreversible changes. To further explore these dynamics, we plan to pharmacologically induce stable microglial activation in future studies. This approach may help clarify the processes underlying glial responses and their impact on neural function. While optical transparency techniques appear to modestly attenuate glial activation, they are insufficient as a standalone solution for long‐term imaging applications.

Studying the real‐time interaction between immune cells and neurons on a large scale remains a significant challenge. This interaction is dynamic and complex, playing a crucial role in maintaining brain homeostasis and orchestrating responses to injury or disease. Immune cells, particularly microglia as the resident macrophages of the central nervous system (CNS), continuously monitor the brain's microenvironment. Upon detecting damage or infection, microglia transition from a surveillant state to an activated phenotype more rapidly than neurons, although the accumulation of pronounced structural remodeling typically occurs over a timescale of hours to days. Other immune cells, including astrocytes, oligodendrocytes, and infiltrating peripheral immune cells such as T cells and macrophages, also contribute by modulating or amplifying the inflammatory response. These interactions occur not only within local brain regions [46] but also exert long‐range effects, bridging central and peripheral systems [47, 48]. Although our study primarily focused on microglial dynamics, the involvement of other cell types in neuroinflammation and recovery following craniotomy should not be overlooked. Astrocytes, the most abundant glial cells in the central nervous system, become activated in response to cerebral insults such as traumatic brain injury. Activated astrocytes play a critical regulatory role by modulating microglial activity through the release of cytokines and chemokines [49]. Moreover, astrocytic endfeet surrounding cerebral vessels are essential for the restoration and maintenance of blood‐brain barrier (BBB) integrity during recovery [50]. In parallel, chemokine release triggered by surgical intervention promotes the recruitment and infiltration of peripheral immune cells, including monocytes, neutrophils, and T lymphocytes. The disrupted BBB further facilitates their entry into the brain parenchyma. These infiltrating immune cells exert dual and context‐dependent functions: while they can exacerbate neuroinflammation and contribute to secondary damage through the secretion of pro‐inflammatory mediators such as MMP‐9, they may also aid in tissue repair by clearing cellular debris and promoting the resolution of inflammation [51]. Taken together, the postoperative recovery process may be better understood within the framework of the neuro‐glial‐vascular unit (GVU), which emphasizes the coordinated interactions among neurons, glial cells, vascular components, and peripheral immune cells as an integrated system [52]. The sequential and dynamic crosstalk among these cellular players—from initial microglial activation to astrocytic response and subsequent peripheral immune cell involvement—drives the transition from acute inflammation to long‐term tissue repair and functional dynamics following craniotomy.

To minimize the number of animals used, the sample sizes in this study (n = 3–4 per group) were kept relatively small. While this may limit the generalizability of our findings and reduce the statistical power to detect more subtle effects, the consistency of the observed results across individual animals, together with the large magnitude of the changes and their statistical significance, provides confidence in our primary conclusions regarding the sequence and timing of post‐craniotomy dynamics. Furthermore, to reduce stress and potential harm to the animals, we did not perform daily recordings throughout the entire 56‐day postoperative period. Instead, we applied linear interpolation to estimate intermediate values between actual measurement points. This approach assumes that postoperative changes occur gradually and approximately linearly between adjacent time points, which is supported by our pilot observations showing smooth temporal trajectories of both microglial activity and behavioral recovery. Linear interpolation has also been widely used in similar longitudinal neuroinflammation studies where continuous sampling is impractical [33, 34, 35]. While increasing the sampling frequency could provide more granular data, the trade‐off in terms of animal welfare and ethical considerations justified our use of interpolation in this study.

To ensure animal welfare, nonsteroidal anti‐inflammatory drug (NSAID) treatment was administered during the first five postoperative days. While this analgesic regimen may partially suppress early neuroinflammatory responses, we still observed a clear and robust sequence of microglial activation and subsequent recovery. This strongly suggests that craniotomy alone is sufficient to induce substantial inflammatory and structural changes. It is plausible that, without NSAID administration, these responses would be even more pronounced. Therefore, our findings likely represent a conservative estimate of the true magnitude of craniotomy‐induced neuroinflammation. Although the standardized NSAID protocol strengthens the reproducibility and comparability of our data, it should be considered a limitation when extrapolating to conditions where postoperative analgesia is absent.

With the development of large‐scale imaging technologies, the ability to monitor glial activation and neuronal function at high spatiotemporal resolution has provided unprecedented insights into the brain's recovery process following surgery. This technological breakthrough enables more comprehensive investigations of dynamic brain states under various conditions, including neurodegenerative diseases, brain injuries, and aging. These advanced imaging tools allow for deeper exploration of the brain's capacity to recover and adapt, opening new avenues for studying neural repair and rehabilitation. Overall, our study not only enhances our understanding of glial activation during post‐surgical recovery but also underscores the critical role of large‐scale imaging in revealing the complex dynamics of brain function and recovery.

## 4 Experimental Methods

### 4.1 Mouse Handling

All animal experiments were approved by the Institutional Animal Care and Use Committee (IACUC) of Tsinghua University (Animal Protocol Number: 17‐DQH1.G25‐1) and conducted in accordance with the National Institutes of Health Guide for the Care and Use of Laboratory Animals. Every effort was made to minimize the number of animals used and their suffering. Mice were housed in the Laboratory Animal Research Centre, maintained at a standard temperature of 24°C, 50% humidity, and subjected to a reversed light cycle, with lights on from 7:00 AM to 7:00 PM.

Microglial cells were visualized using Cx3cr1‐GFP transgenic mice (JAX #005582), which express green fluorescent protein (GFP) linked to the Cx3cr1 gene, a key receptor for microglial function. Neuronal structure was examined in Thy1‐YFP transgenic mice (JAX #003709), where a subset of neurons expresses yellow fluorescent protein (YFP), enabling clear visualization of neuronal morphology. For imaging neuronal activity, we utilized a cross between Rasgrf2‐2A‐dCre mice (JAX #022864) and Ai148(TIT2L‐GC6f‐ICL‐tTA2)‐D mice (JAX #030328), which express the GCaMP6f indicator in layer 2/3 cortical neurons. Experimental mice were intraperitoneally injected with 0.25 mg/g trimethoprim (TMP, Sigma) for two consecutive days [53]. Healthy adult mice (20–30 g, 8–24 weeks old), both male and female, were used in the experiments. Prior to surgery, mice had access to food and water ad libitum. After surgery, they were housed individually for the duration of the experiments. Following craniotomy, each mouse was included in long‐term imaging studies.

Craniotomy Operation for in vivo Imaging: The craniotomy procedure was performed as previously described [54]. Briefly, mice were first anesthetized with isoflurane (3% induction, 1.5% maintenance) and placed in a stereotaxic frame to ensure proper positioning during surgery. The skin over the skull was carefully incised, and the underlying bone was exposed. A small cranial window was then created over the targeted cortical region. A piece of the skull was carefully removed, and a sterile glass coverslip (diameter = 7 mm) was used to replace the excised bone. This coverslip was securely affixed to the skull using Krazy Glue (Elmer's Products Inc.). Finally, a head‐post was attached to the edge of the skull with a layer of dental acrylic to provide stability for subsequent imaging procedures. Flunixin meglumine (1.25 mg/kg, Sichuan Dingjian Animal Medicine Co.), an anti‐inflammatory drug, was administered subcutaneously for five consecutive days post‐surgery. This step complied with institutional ethical requirements and minimized animal pain and distress. Postoperative analgesia was provided using a NSAID, which was administered at a standardized dose and fixed schedule immediately after each imaging session to ensure consistency across all animals and reduce variability in postoperative recovery. This standardized approach provided a controlled baseline for subsequent assessment of microglial dynamics and behavioral outcomes.

After craniotomy, mice were allowed to recover from anesthesia. Then, they were subjected to 2pSAM (morphology) [18] or RUSH 3D (neural activity) [25] imaging. Mice not in good health during recovery were euthanized and excluded from the study. Only those that completed the full imaging protocol were included in the data analysis.

### 4.2 2pSAM Imaging for Cell Morphology

We employed a custom‐built two‐photon synthetic aperture microscopy (2pSAM) [18] system to obtain the morphology of microglia and also neurons after craniotomy surgery. In brief, 2pSAM replaces high‐NA point excitation with needle‐like, low‐NA sub‐aperture beams that are swept across multiple angles and then computationally synthesized into a high‐resolution 3D volume via tomographic deconvolution. A small pinhole at the intermediate focus provides a ptychographic spatial constraint so that incoherent fluorescence projections from different angles can be combined up to (near) the whole‐objective NA limit. Critically, this scheme extends depth of field for each shot, uses lower peak intensity (reducing nonlinear photodamage), and enables digital aberration correction (DAO) without extra wavefront hardware by inferring sub‐aperture phase gradients from lateral PSF shifts; DAO is applied tile‐wise for multisite correction. Excitation was provided by an 80 MHz femtosecond laser (InSight X3) at 920 nm for standard imaging and 800 nm for laser‐induced injury. A 25×/1.05 NA water‐immersion objective (Olympus XLPLN25XWMP2) was used. Fluorescence was collected through four PMTs with standard dichroic mirrors and emission filters for simultaneous multicolor detection. Each volume consisted of 512 × 512 pixels acquired from 13 angular sub‐aperture positions. The net volumetric imaging rate was approximately 2.3 volumes/s, increasing to 30 volumes/s with sliding‐window reconstruction and motion correction. It achieves minimally invasive, high‐speed, and aberration‐corrected 3D imaging of subcellular dynamics at a millisecond scale over large volumes in deep tissue, with reduced phototoxicity to enable challenging continuous long‐term in vivo imaging applications in mice. For the 2pSAM system, multi‐angle diffraction coding based on spatial constraints was proposed to achieve incoherent optical aperture synthesis. Two‐photon synthetic aperture microscopy was established, “turning points into needles.” Through the scanning of multi‐angle needle‐shaped beams, high‐speed 3D perception is achieved simultaneously. We employed a 4 × 4 scanning collection approach to efficiently capture cell information across a large field of view.

### 4.3 Immunofluorescence Staining and Confocal Imaging

Thy1‐YFP Transgenic mice were deeply anesthetized via intraperitoneal injection of Avertin at a dose of 250 mg/kg body weight, and transcardially perfused with 25 mL ice‐cold phosphate‐buffered saline (PBS) and 25 mL 4% paraformaldehyde (PFA). Brain tissue was then post‐fixed in 4% PFA at 4°C overnight, followed by cryoprotection in graded 20 and 30% sucrose solutions. The brains were sectioned in the coronal plane at a thickness of 15 µm using a cryostat (CM1950, Leica). Coronal sections (2.00 mm posterior to bregma) were first incubated in 0.3% Triton X‐100 solution for 15 min (P0096, Beyotime) and then in immunostaining blocking buffer for 15 min (P0260, Beyotime). Next, sections were incubated with primary antibody (IBA1, 1:200, ab178846, Abcam) at 4°C overnight. After rinsing three times in PBS, the sections were incubated with the corresponding secondary antibody for 1 h at room temperature. Finally, sections were mounted using Prolong Diamond Antifade Mountant (P36961, Invitrogen) with DAPI, and scanned under a confocal microscope (FV1000, Olympus).

### 4.4 RUSH 3D Imaging for Neural Activities

The RUSH 3D system was described in previous work [25]. In brief, RUSH 3D is a compact scanning light‐field mesoscope that measures full 4D light fields (space × angle) with a coded microlens array (MLA) on a 48‐MP CMOS camera, then reconstructs high‐fidelity 3D volumes computationally. The system integrates: Wave‐optics Digital Adaptive Optics (wDAO) to correct strong, spatially varying system and tissue aberrations (validated up to ∼6λ RMS), tile‐by‐tile, without slowing acquisition; Multiscale Background Rejection (MBR) for computational optical sectioning (suppresses low‐frequency out‐of‐focus/background); and A temporal sliding window to keep 3D frame rate near the camera's limit. This yields uniform ∼2.6 × 2.6 × 6 µm^3^ resolution over 8 × 6 × 0.4 mm^3^ at 20 Hz, with low phototoxicity, in a compact, off‐the‐shelf build. Volumes of 8 × 6 × 0.4 mm^3^ were acquired at 20 Hz with ∼2.6 × 2.6 × 6 µm^3^ resolution using a temporal sliding window. Reconstruction comprised pixel realignment, wDAO (tile‐wise wave‐optics DAO) and MBR, with time‐weighted motion correction. It is a novel imaging method that integrates multiple computational imaging techniques within a scanning light‐field framework. This approach enables long‐term, high‐speed, centimeter‐wide mesoscale 3D imaging at single‐cell resolution. It operates within a compact system, making it suitable for a wide range of practical applications. The method is particularly useful for studying large‐scale intercellular dynamics at the mammalian organ level, offering significant advancements in both spatial and temporal resolution.

### 4.5 Visual Stimuli System

Visual stimuli were presented on an LCD monitor positioned to the left eye [55]. To align the tangent point between the monitor's plane and the sphere surrounding the right eye, the center of the screen was set at the eye's focal point. The monitor was fixed at a 30° counter clockwise angle relative to the antero‐posterior axis of the mouse and tilted 20° toward the animal along the gravitational axis. The distance from the monitor's center to the right eye was maintained at 5 cm. The display covered a horizontal field of view of 117° and a vertical field of view of 110°.

Stimuli were generated and controlled using the Psychophysics Toolbox in MATLAB [56]. The spatial frequency was set to 0.1 cycles per degree, with a temporal frequency of 2 cycles per second. Full‐screen gratings were shown for 2 s, followed by an 8–10 s interstimulus interval. To induce population responses across different visual stimulus orientations, 4 stimuli were used, oriented at 0°, 45°, 90°, and 135°, presented in a random order.

### 4.6 Synchronization of Imaging and External Stimulation

To synchronize brain imaging with visual stimuli, trial‐specific signals were generated and recorded using the digital or analog inputs of the Omniplex system. A trigger from the RUSH 3D camera was routed to the digital input for precise timing. During visual stimulation, a small square in the bottom right corner of the screen changed color from white (indicating the start) to black (indicating the end). This color shift was detected by a photodiode, which then sent transistor‐transistor logic (TTL) signals to the digital input interface of the Omniplex system to mark the stimulus onset and offset.

### 4.7 Sholl Analysis of Microglial Morphology

Sholl analysis was performed to quantify the morphological complexity of microglia. In brief, microglia with clearly visible soma and primary processes were randomly selected by a blinded researcher. A series of concentric circles were established from the soma extending in steps of 2 µm to the most distal glial projections. The maximum Sholl radius (Max number of intersections) and total number of intersections (Area under curve) were applied as primary metrics for morphological complexity. A total of 30 cells per mouse at each timepoint were analyzed to ensure robust statistical power by using Fiji software with the Neuroanatomy/Sholl plugin.

### 4.8 Neuron Extraction Pipeline for RUSH 3D System

Given the high throughput of the RUSH 3D system, we developed a tailored parallel data analysis pipeline based on Constrained Nonnegative Matrix Factorization for microEndoscopic (CNMF‐E) [57]. It is an algorithm designed to extract the activity of individual neurons from calcium imaging data, particularly from single‐photon microendoscopic recordings. CNMF‐E improves signal extraction by separating neuronal signals from background noise and motion artifacts, thereby enabling more accurate and reliable identification of neural activity. In brief, the raw video data were first registered to correct for motion artifacts, followed by temporal summarization into pixel‐neighbor correlation and peak‐to‐noise ratio images. The Hadamard product of these images was used to initialize neuronal candidates. Neuronal footprints and temporal activities were further refined using constrained non‐negative matrix factorization with a ring model to account for background noise. To exclude neurons located on blood vessels, we generated an intensity‐based vessel segmentation mask. Additionally, the extracted temporal signals underwent filtering through a supervised deep neural network, trained via manual inspection, to eliminate false signals caused by motion artifacts or hemodynamic interference. Finally, the neuronal footprints were projected onto a visual response‐intrinsic map within the Allen Mouse Brain Common Coordinate Framework (CCF v3) [58], enabling the localization of each neuron within specific cortical areas.

### 4.9 Neuronal Function Analysis

We investigated the functional recovery dynamics of neurons by analyzing their stimulus responses and functional connectivity. For each angle of raster stimulation, we established stimulus‐responsive neuronal populations by comparing means and standard deviations to identify neurons that showed significant activation during the stimulus window (∆t = 2s) compared to pre‐stimulus trace (Figure 5C–E). Specifically, a neuron was considered responsive during a given stimulus window if its mean activation intensity during the stimulation period exceeded the baseline mean activation (pre‐stimulus interval) plus three standard deviations of the baseline activity.

By aligning the spatial coordinates of these neurons with the standard Allen Brain Atlas, we present the spatial distribution of stimulus‐responsive neurons across cortex (Figure 5F) and quantified the number of neurons in each brain region (Figure 5G). The neuronal population within each brain region was temporally normalized to delineate distinct dynamic evolutionary patterns across regions (Figure 5H).

Decoding accuracy was quantified as the performance of a support vector machine (SVM) in classifying neuronal responses to drifting gratings into one of four orientations (Figure 6A). The decoding accuracy is defined as the ratio of correctly classified trials to the total number of trials, that is,

(1) $$Acc=\frac{N\left(Correct\;Trials\right)}{N\left(Total\;Trials\right)}$$

where *N* indicates the number of trials. A lower accuracy in this task indicates a reduced discriminability of visual information by the neuronal population and, consequently, diminished visual function in mice

Functional connectivity was assessed by calculating the Pearson correlation between the activities of different units (Figure 6C–E). At the neuronal level, we computed the Pearson correlation coefficients between all stimulus‐responsive neuron pairs to construct a cortex‐wide functional network, that is,

(2) $$Connectivity_{x,y}=\frac{\sum_{i=1}^{n}\left(x_{i}-\bar{x}\right)\left(y_{i}-\bar{y}\right)}{\sqrt{\sum_{i=1}^{n}{\left(x_{i}-\bar{x}\right)}^{2}\sum_{i=1}^{n}{\left(y_{i}-\bar{y}\right)}^{2}}}$$

where *x_i_* and *x_i_* are the *i^th^* observed values of sample x and y, *x_i_* and *x_i_* indicate the average values of sample x and y. High functional connectivity between a pair of neurons (e.g., neurons x and y) indicates strong correlation in their neural response activities. At a systems level, high global functional connectivity across brain regions reflects a robust capacity for large‐scale neural coordination and integrated brain function. The weight distributions of all functional connections within the network were presented across postoperative days (Figure 6C). We calculated the network‐level metrics, included the global mean correlation coefficient (Figure 6D) and strong connection ratio (Figure 6E). The strong connection ratio was defined as the proportion of connections with correlation coefficients above 0.8 relative to all existing connections.

At the brain region level, neuronal activity within each region was first averaged to obtain the average response trace, followed by the calculation of the Pearson correlation between the average activities of each region (Figure 6F). Only the functional connectivity between brain regions (N = 19) that persisted for several days post‐surgery is presented here. The dashed rectangle in Figure 6F delineates the clustering results of the functional connectivity matrix on postoperative day 1, computed using spectral clustering algorithm (sklearn.cluster.SpectralClustering in python).

Modularity measured the strength of division of a network into modules. To assess the modular structure of functional connectivity, we calculated the network modularity coefficients (Figure 6G), defined as

(3) $$Q=\frac{1}{2m}\sum_{ij}\left(A_{ij}-\gamma \frac{k_{i}k_{j}}{2m}\right)\delta \left(c_{i},c_{i}\right)$$

where *m* is the number of edges (or sum of all edge weights), *A* is the adjacency matrix of the network, *k_i_* is the weighted degree of *i*, γ is the resolution parameter, and δ(*c_i_*,*c_i_*) is 1 if i and j are in the same community else 0. A higher modularity value indicates a more distinct community structure, characterized by dense intra‐community connections and sparse inter‐community links.

Degree centrality quantifies a node's number of connections (its degree) relative to the rest of the network (Figure 6H). Specifically, it is defined as the number of edges incident to a node. The metric is typically calculated using the formula:

(4) $$degree\;centrality\;\left(v\right)=\sum_{u}k\left(v,u\right)$$

where *k*(*v*, *u*) denotes connection weight between node *v* and node *u*. This metric assesses the significance of each node (i.e., brain region) as an interaction hub, enabling comparisons of importance across different days and brain regions. A node with higher degree centrality possesses greater potential influence or importance in the functional network. This metric assesses the significance of each node (i.e., brain region) as an interaction hub, enabling comparisons of importance across different days and brain regions.

### 4.10 Statistical Analysis

Data were analyzed using Python and GraphPad Prism (v8.0, GraphPad Software). The basic statistical information of data is presented as mean ± standard deviation (mean ± s.d., e.g., Figure 3D) or mean ± standard error of the mean (mean ± s.e.m, e.g., Figure 5E). Individual data points are displayed as scatter (e.g., Figure 4C) or line (e.g., Figure 6D) plots. In the functional analysis parts, we determine the average of the baseline values across all biological replicates, then normalize the rest of the values to that average. 1) Baseline Calculation: The mean value of day 1 data across all biological replicates was used to represent the baseline, allowing for inter‐animal variability. This will provide variability in the baseline data, which is essential for comparison. 2) Data Normalization: For each subsequent time point (day 4, day 7, day 14, etc.), normalize the data by dividing the values by the baseline average. This will allow meaningful comparisons between time points.

In the statistical analysis, the *Shapiro‐Wilk* test was first applied to confirm the normality of the data. *F*‐test was used to compare variances between different groups. For longitudinal comparisons within the same animal, repeated measures (RM) one‐way ANOVA followed by Tukey's *post hoc* test was applied when data met normality assumptions; otherwise, the Friedman test with Dunn's *post hoc* correction was used. For comparisons between independent groups, an unpaired one‐way ANOVA with Tukey's *post hoc* test was employed. *Pearson* correlation analysis was performed to measure the relation between two groups. The Source Data file provides comprehensive statistical details, for example, the rank sum difference and p‐value for the Friedman test with Dunn's *post hoc* correction, and the 95.00% confidence interval (CI) of the difference and p‐value for the RM one‐way ANOVA followed by Tukey's *post hoc* test.

The statistical results of each experiment are provided in the corresponding figure legends, including the sample size (n), statistical values and significance annotations (e.g., *p < 0.05, **p < 0.01, ***p < 0.001, ****p < 0.0001, ns – not significant). P‐values less than 0.05 were considered as statistically significant differences.

## Author Contributions

This project was the result of a close collaboration. G. Xiao, J. Cao, L. Li, and D. Yao conceived the idea of this project. G. Xiao designed the detail experiments in each session. Z. Wang and D. Yao performed all the mouse experiments, with the assistance of J. Xie, P. Zheng, Y. Wang, Y. Chen, Z. Wang, Q. Ji, C. Gao, and A. Li. L. Li analyzed the neuronal response to visual stimulus with discuss with G. Xiao. D. Yao, L. Li, and Z. Wang made figures with discuss with G. Xiao and M. Wang. G. Xiao, L. Li, D. Yao, and Z. Wang wrote the manuscript with feedback from all the authors. G. Xiao revised and polished the paper. G. Xiao and J. Cao supervised all aspects of the project.

## Funding

The authors have nothing to report.

## Conflicts of Interest

The authors declare no conflicts of interest.

## Supporting information




**Supporting File 1**: advs74382‐sup‐0001‐SuppMat.docx.

**Supporting File 2**: advs74382‐sup‐0002‐VideoS1.mp4.

**Supporting File 3**: advs74382‐sup‐0003‐VideoS2.mp4.

## Data Availability

The calcium imaging raw data supporting the findings of this study exceed 10 TB in size. To facilitate access, code for data analysis involving neural activities from this work is freely available online: https://github.com/IrisRegion/Post‐Craniotomy‐Project.git. The raw dataset is available from the corresponding authors upon reasonable request.
