# Supplementary material for: Mesoscale Recovery of Microglial and Neuronal Dynamics After Craniotomy Across Wide Cortex in Transgenic Mice
Source: Adv Sci (Weinh). 2026 Feb 11;13(23):e12192. doi: 10.1002/advs.202512192 (PMC13104116; doi:10.1002/advs.202512192)
Supplement: Supplementary file 1 — Supporting File 1: advs74382‐sup‐0001‐SuppMat.docx. [file ADVS-13-e12192-s001.docx]

Supporting Information

Mesoscale Recovery of Microglial and Neuronal Dynamics after Craniotomy across wide Cortex in Transgenic Mice

*Guihua Xiao, Zhilei Wang, Pengchang Zheng, Jingyu Xie, Yangzhen Wang, Yun Chen, Zilin Wang, Ao Li, Minghuan Wang, Quanbo Ji, Can Gao, Di Yao, Lingbo Li, Jiangbei Cao*


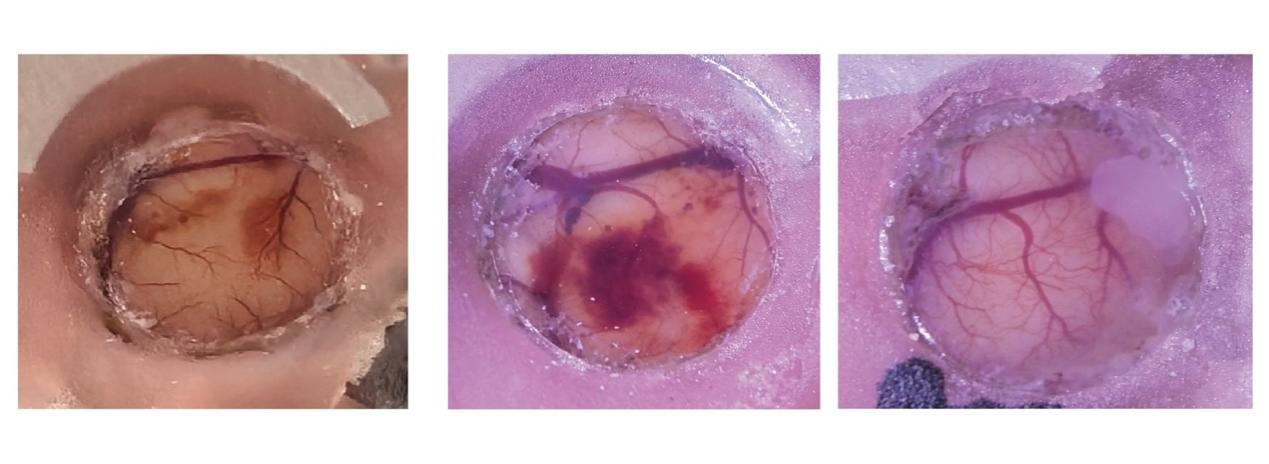


**Figure S1.** Representative examples of unsuccessful craniotomies due to postoperative complications, including varying degrees of bleeding (from mild (Left) to severe hemorrhage (Middle)), and infections along the cranial suture (Right). Because such conditions could potentially influence cranial window recovery and subsequent tissue responses, only animals with a clear, infection-free, and hemorrhage-free cranial window were included in the final analyses. Animals exhibiting any of these complications were excluded from quantitative data.

**
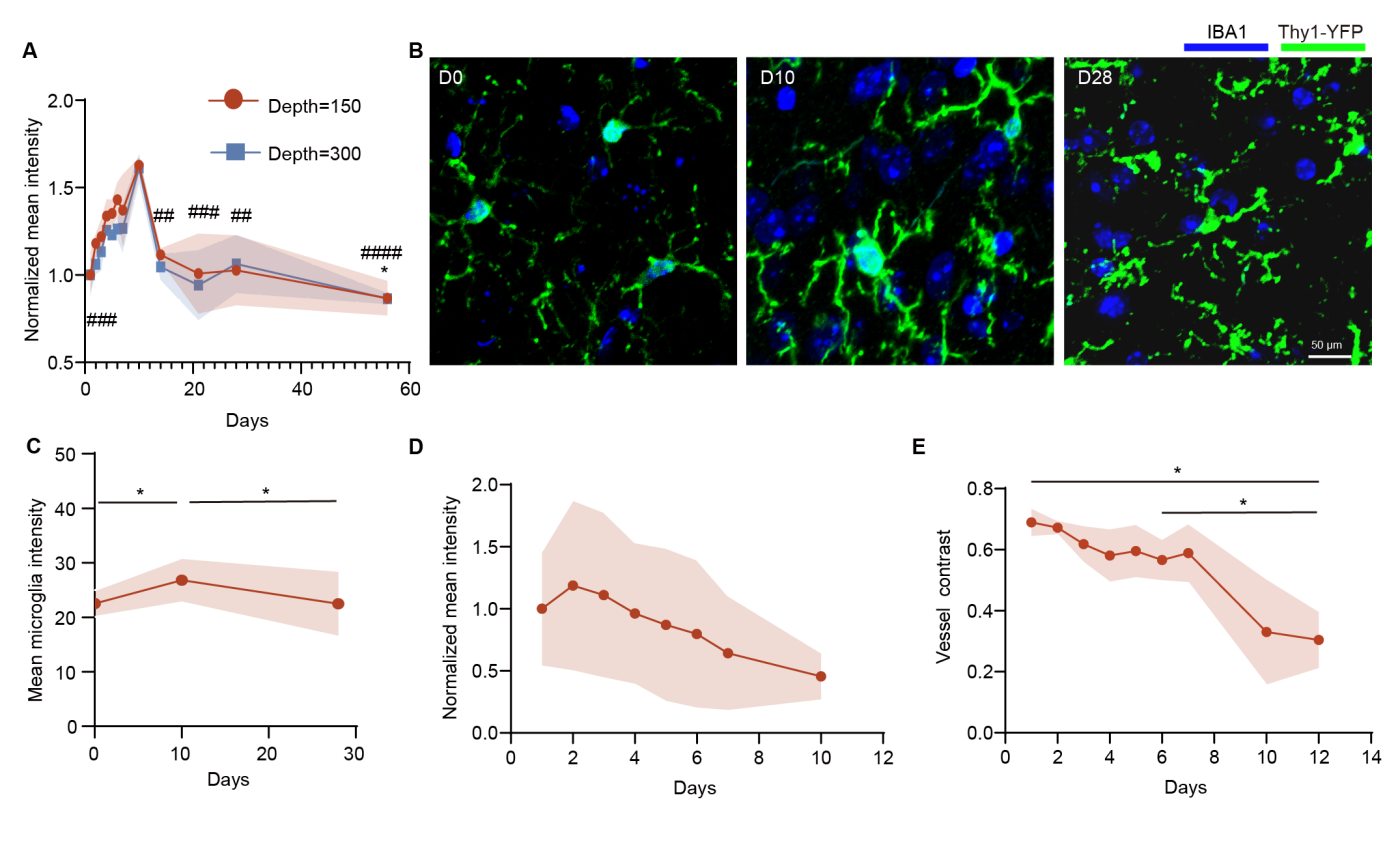
**

**Figure S2.** Statistical analysis of long-term microglial dynamics following craniotomy. (A) Quantitative analysis of the normalized mean intensity of microglia from 2D image projections. (n=3 mice) (B) Representative images of microglia, immunolabeled for the microglial marker Iba1 in *ex vivo* brain sections, from the cortex ipsilateral to the surgical window at baseline (day 0), day 10, and day 28 post-craniotomy. Scale bar = 50 μm. (C) Quantitative analysis of microglial mean intensity derived from the data shown in panel (B). (n=6 mice for each group). (D) Quantitative analysis of the normalized mean intensity of microglia following skull thinning and optical clearing procedures. (n=3 mice). (E) Quantification of cranial bone regeneration, assessed by using vessel contrast as the index. (n=3 mice). Friedman test with Dunn's *post hoc* correction in (A). *P < 0.05 vs Peak-timepoint for depth =150 μm, and ##P < 0.01, ###P < 0.001, ####P < 0.0001 vs Peak-timepoint for depth =300 μm in (A). Unpaired One-way ANOVA with Tukey’s *post hoc* correction in (C). RM One-way ANOVA, Tukey’s *post hoc* correction in (D and E). *P < 0.05.


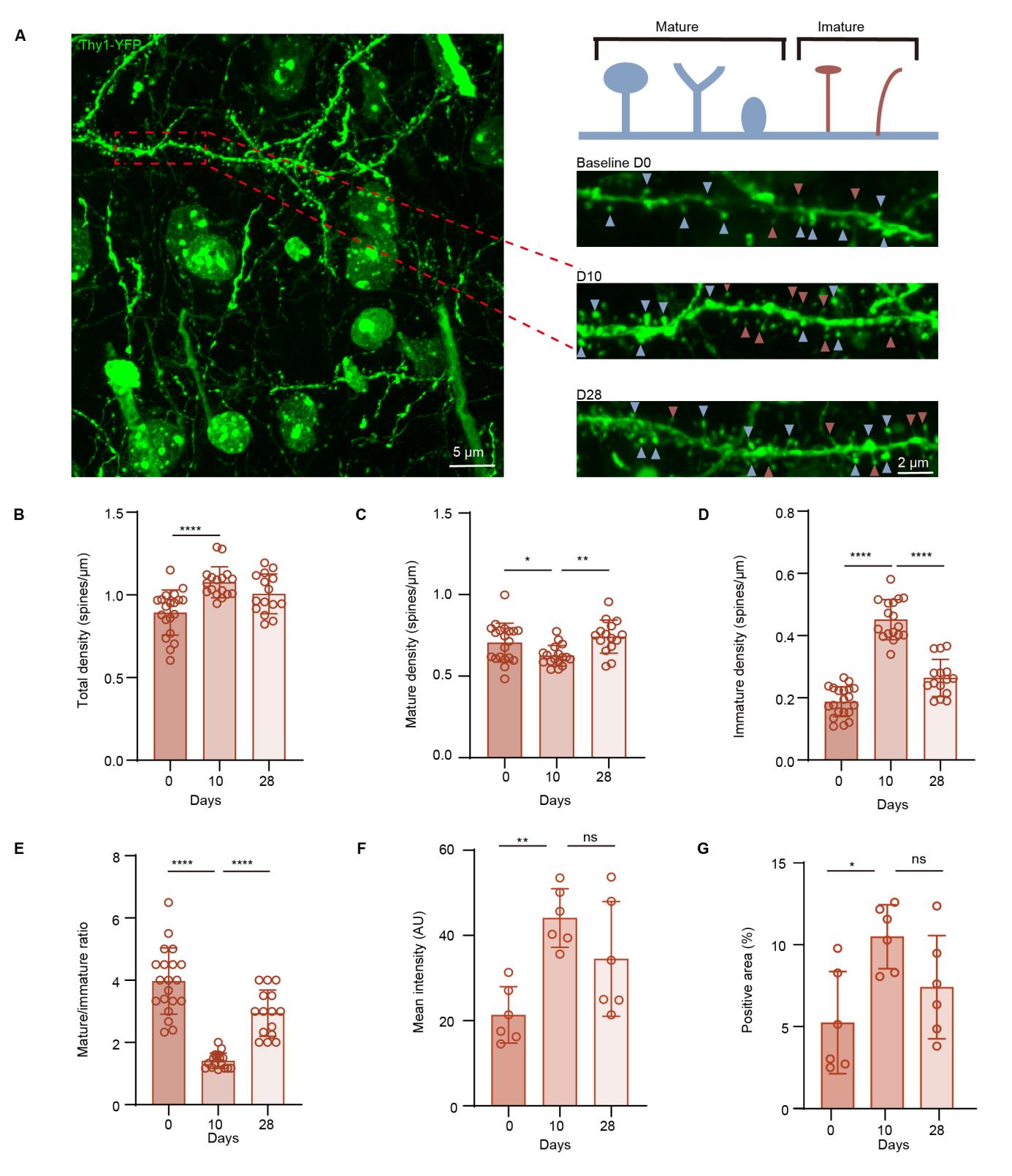


**Figure S3.** Statistical analysis of long-term dendritic spine dynamics following craniotomy. (A) Representative images of dendritic spines in the cortex ipsilateral to the cranial window at baseline, day 10, and day 28 post craniotomy. Blue arrows indicate mature spines, and orange arrows indicate immature spines. (B-E) Quantitative analysis of total dendritic spine density (B), mature spine density (C), immature spine density (D), and the ratio of immature to total spines (E) over time. *P < 0.05, **P < 0.01, ****P < 0.0001. n = 15 from 6 mice. (F-G) Quantitative analysis of Thy1-YFP labeled spine intensity and positive area. **P < 0.01. n=6 mice. Unpaired One-way ANOVA with Tukey’s post hoc correction in (B-G).
